# Towards high performance computing for molecular structure prediction using IBM Cell Broadband Engine - an implementation perspective

**DOI:** 10.1186/1471-2105-11-S1-S36

**Published:** 2010-01-18

**Authors:** SPT Krishnan, Sim Sze Liang, Bharadwaj Veeravalli

**Affiliations:** 1 Institute for Infocomm Research, 1 Fusionopolis Way, #21-01, Connexis South Tower, Singapore 138632; 2 Department of Electrical and Computer Engineering, National University of Singapore, 4 Engineering Drive 3, Singapore 117576

## Abstract

**Background:**

RNA structure prediction problem is a computationally complex task, especially with pseudo-knots. The problem is well-studied in existing literature and predominantly uses highly coupled Dynamic Programming (DP) solutions. The problem scale and complexity become embarrassingly humungous to handle as sequence size increases. This makes the case for parallelization. Parallelization can be achieved by way of networked platforms (clusters, grids, etc) as well as using modern day multi-core chips.

**Methods:**

In this paper, we exploit the parallelism capabilities of the IBM Cell Broadband Engine to parallelize an existing Dynamic Programming (DP) algorithm for RNA secondary structure prediction. We design three different implementation strategies that exploit the inherent data, code and/or hybrid parallelism, referred to as C-Par, D-Par and H-Par, and analyze their performances. Our approach attempts to introduce parallelism in critical sections of the algorithm. We ran our experiments on SONY Play Station 3 (PS3), which is based on the IBM Cell chip.

**Results:**

Our results suggest that introducing parallelism in DP algorithm allows it to easily handle longer sequences which otherwise would consume a large amount of time in single core computers. The results further demonstrate the speed-up gain achieved in exploiting the inherent parallelism in the problem and also elicits the advantages of using multi-core platforms towards designing more sophisticated methodologies for handling a fairly long sequence of RNA.

**Conclusion:**

The speed-up performance reported here is promising, especially when sequence length is long. To the best of our literature survey, the work reported in this paper is probably the first-of-its-kind to utilize the IBM Cell Broadband Engine (a heterogeneous multi-core chip) to implement a DP. The results also encourage using multi-core platforms towards designing more sophisticated methodologies for handling a fairly long sequence of RNA to predict its secondary structure.

## Background

In this section, we begin by presenting the relevant biological background behind RNA and then give a brief summary on IBM Cell platform; Astute readers are referred to [1,2] for further details. Following this, we describe RNA secondary structure prediction from a dynamic programming perspective and list related works. Finally, we analyze a Dynamic Programming (DP) algorithm for RNA secondary structure prediction.

### RNA

A ribonucleic acid (RNA) sequence is made of individual molecules known as nucleic acids, which can be one of four possible types - Adenine (A), Cytosine (C), Guanine (G), and Uracil (U) [3]. A RNA sequence folds upon itself to form bonds among its molecules known as base pairs and the overall structure is then referred as its secondary structure.

There are several types of patterns that make up the RNA secondary structure - loops (hairpin, internal), stems, bulges, free dangling ends and pseudoknots. In particular, pseudoknots are of prime interest for they play an important role in regulatory, catalytic, messaging and structural activities in cells. Therefore, elucidation of structural features of pseudoknots and reliable prediction of pseudoknots in RNA secondary structure using sequence data are important for understanding structure-function relationships in many RNA molecules.

**Figure 1:**
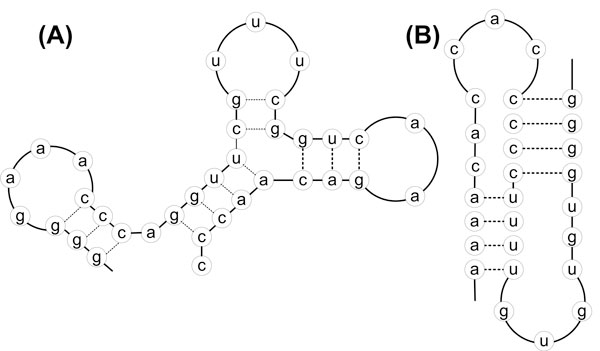
**RNA secondary structures - A: Non-Pseudoknots, B: Pseudoknots**. This figure illustrates pictorially RNA secondary structure in both Pseudoknot and Non-pseudoknot configurations.

Figure 1 shows an example of RNA secondary structure in both Pseudoknot and Non-Pseudoknot configurations.

### IBM Cell

Cell is a heterogeneous RISC-based multi-core microprocessor architecture jointly developed by Sony, Toshiba and IBM. Cell contains a 64-bit PowerPC based 2-way processor core with SIMD extensions, known as the PowerPC Processing Element (PPE). There are 8 co-processors known as Synergistic Processing Elements (SPE) that are optimized for mathematical computations. A high-bandwidth circular data bus known as the Element Interconnect Bus (EIB) interconnects the PPE and SPEs.

Each SPE contains 256 KB local memory and no cache memory. The SPU can only access data from the local memory, and uses DMA to fetch data from the main memory. Therefore, SPE programs have to pre-fetch data of optimal sizes and leverage on locality of reference for best performance. Langou et al [4] demonstrated a 3.2 GHz Cell with 8 SPEs delivering a performance equal to 100 GFLOPS on an average double precision Linpack 4096 × 4096 matrix. SPEs are the work-horses while the PPE handles the control and coordination of the tasks.

**Figure 2:**
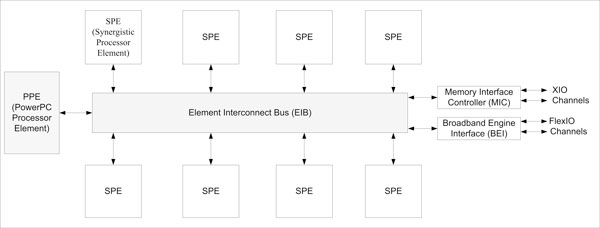
**Cell schematic**. This figure shows the IBM Cell Broadband Engine processor schematic. Various elements of the chip such as PPE, SPE, EIB are shown.

Figure 2 shows a simplified layout of the Cell Broadband Engine.

In this paper, we used Sony PS3 game console for our implementation. It may be noted that Sony PS3 game console uses the IBM Cell processor. In Sony PS3, only six of eight SPEs are available to the programmer. The specifications of the Sony PS3 are presented below in Table 1.

**Table 1:** PS3 hardware specifications. This table shows the various hardware components of the Sony Play Station 3 (PS3) game console with details on size of each of them

CPU Architecture	PPE: PowerPC 64 bit SPE: SPE Architecture
Clock Speed	PPE: 3.2 GHz SPE (each): 3.2 GHz

L2 Cache (PPE Only)	512 KB

PPE Memory	256 MB XDR

Memory Bandwidth	25.6 GB/s

SPE Memory	256 KB SRAM

Single Precision	PPE: 25.6 GFLOPS SPE: 25.6 GFLOPS

Double Precision	PPE: 6.4 GFLOPS SPE: 14 GLOPS

### Dynamic Programming

RNA secondary structure prediction is the process of determining the optimal set of pairings between nucleotides. One of the well known computational methods for RNA secondary structure prediction is dynamic programming, which evolves a final optimal structure by computing best solutions for overlapping sub-problems. In its simplest form the algorithm performs base-pair maximization.

**Figure 3:**
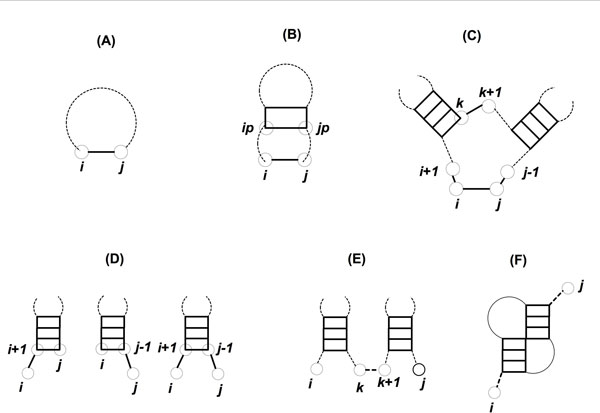
**RNA secondary structure building blocks**. This figure shows the various types of building blocks that form the RNA secondary structure. 3A shows the hairpin loop, 3B shows stack/bulge/internal loop, 3C shows bifurcation loop, 3D shows the three configurations of free dangling ends, 3E shows the open bifurcation and 3F shows the pseudoknot.

The optimal structure between two nucleotide positions can be derived recursively from the optimal substructures contained within. Dynamic programming works from the smallest substructure upwards, storing intermediate results along the way for future back-referencing. Dynamic programming for RNA structure prediction is classified as non-serial polyadic [5]. This means that the optimal structure at any stage has data dependency on the results of previously calculated substructures, not necessarily derived during the previous iteration. The optimal structure for any sub-sequence involves considering all the possible pairings that could take place amongst nucleotides contained in that particular sub-sequence. The possibilities are shown in Figure 3.

**Figure 4:**
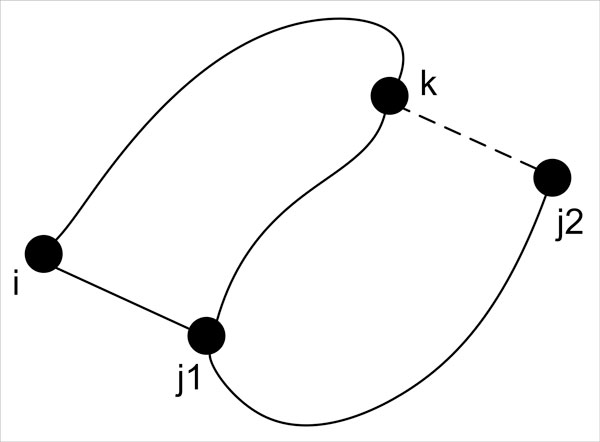
**Pseudoknot from a dynamic programming perspective**. This figure shows how a pseudoknot is modeled using the dynamic programming paradigm.

Scenario F in the Figure 3 is the pseudoknot case which needs more elaboration. Figure 4 shows a typical pseudoknot from a dynamic programming perspective.

In Figure 4, in order for the dynamic programming algorithm to compute (i, j1), it would no longer be sufficient to look within sub-sequences of (i, j1) as for every position k within (i, j1), there is a need to assess the implication of k paring with some nucleotide at any position j2 outside of (i, j1). The complication is that to assess the effect of (k, j2), it also depends on how every j1 within (k, j2) might pair with some nucleotide at any position i outside of (k, j2).

### Related work

Several DP and non-DP based algorithms have been published in the literature. Zuker and Stiegler proposed a simple DP for predicting optimal secondary structures without pseudoknots [6]. The algorithm used thermodynamic parameters as heuristics, has a time complexity of O(n^3) and is implemented in mfold software [6].

Alternative methods to DP, especially to reduce the complexity introduced by pseudoknot prediction, were explored. In particular methods such as quasi-Monte Carlo search, genetic algorithms, Hopfield networks and stochastic context-free grammars [3] had limited success in specialized scenarios.

Akutsu reformulated an approach based on tree-adjoining grammar (by Uemura et al) into a DP procedure, and presented a DP solution for secondary structure prediction with simple pseudo-knots. The algorithm had O(n^4) complexity, and was based on base-pair maximization [7]. The DP solution was easier to understand, modify and maintain compared to Stochastic Context-Free Grammar (SCFG) based algorithms.

Jitender et al extended Akutsu's solution to incorporate thermodynamic parameters in order to predict simple pseudoknots in optimal RNA secondary structures [3]. They used thermodynamic parameters derived and provided by Zuker, Mathews and Turner [8] and retained the time complexity of O(n^4).

Independently, Rivas and Eddy had actually proposed a different DP approach to the pseudo-knot problem and implemented in pknots [9]. The algorithm is non-trivial and predicts general pseudoknots in thermodynamically optimal secondary structures.

It is difficult to develop generic parallel algorithms on multi-core architectures due to unique data dependencies, memory access methods and load balancing [10]. Tan Sun and Gao identified small local memories and increasing number of cores as a trend in multi-core design and suggested that DP for multi-cores be designed for data transformation and finer grain parallelism for better performance [10].

### Analysis of PKNOTS

Rivas, E. developed a dynamic programming algorithm for predicting optimal secondary structures with general pseudoknots by extending the work of Zuker et al. and by using thermodynamic parameters [9]. The algorithm is implemented in pknots [9].

**Figure 5:**
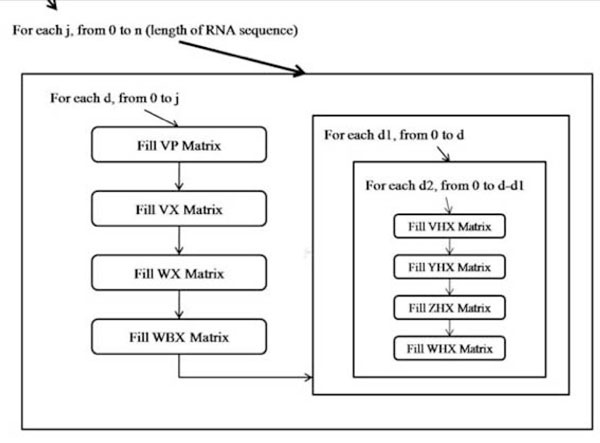
**Nesting relationship among matrix-filling functions**. This figure shows the control flow and sequence of operation in a dynamic programming algorithm as time passes and the various matrices that are populated depending on the input sequence length.

The prediction of the secondary structure involves iterating through all possible sub-sequences of increasing lengths, and computing their respective optimal structures. Figure 5 describes the process. The intermediate results are stored in two 2-dimensional matrices, traditionally named VX and WX, to compute the optimal configuration with the assertion that (i, j) itself pairs, and the general optimal configuration with no assertion. Notice that VX would have to be calculated before WX, and that VX corresponds to scenarios A, B and C in Figure 3, while WX includes the possibilities of scenario D, E and F.

Rivas, E. introduced the idea of one-hole gap matrix to compute the pseudo-knot scenario. This introduces the need for four 4-dimensional matrices, over and above the original VX and WX 2-dimensional matrices. The scalar implementation uses two more 2-dimensional matrices called WBX and VP for holding auxiliary information about the WX and VX matrices.

Table 2 below illustrates the data dependencies between matrices, which is equivalent to the data dependencies between the matrix filling functions. The data dependency is from row to column.

**Table 2:** data dependencies in PKNOTS algorithm. This table describes the dependency relationship among the various matrices in the dynamic programming (DP) algorithm

	VP	VX	WX	WBX	VHX	ZHX	YHX	WHX
VP	O	O						

VX	O	O		O		O	O	O

WX		O	O				O	O

WBX		O		O			O	O

VHX					O			O

ZHX		O		O	O	O		O

YHX		O		O	O		O	O

WHX		O		O	O	O	O	O

The crucial note is that the data dependencies are both non-serial and polyadic. By non-serial, we refer to the fact that loop iterations are dependent not only on the previous iteration, but also on the many previous iterations. Polyadic because the data dependency is one-to-many. In addition, all of the matrix filling functions reference thermodynamic data from a matrix of size 4276 × 4276.

The algorithm, with four 4-dimensional matrices and four 2-dimensional matrices, has a space complexity of O(n^4^), dominated by the space requirement of a 4-dimensional matrix. The time complexity of the implementation, from the layout, and described by Rivas, E. in [9], is O(n^6^).

## Methods

In this section we will describe our parallelization efforts to port pknots algorithm to the IBM Cell platform. Sections 2.1 - 2.5 describe the 3 parallel implementations for pknots and 2 serial implementations for performance comparisons.

In our quest to build the best possible parallel version, we attempted various strategies and exploited several relationships among the code graphs and data paths of the pknots scalar implementation. We begin by analyzing the scalar implementation to identify suitable locations in the algorithms for introducing parallelization.

**Figure 6:**
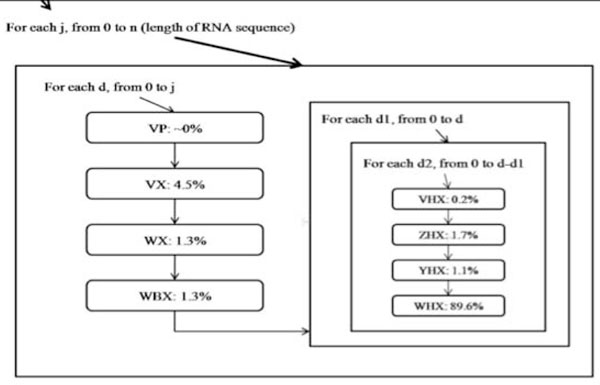
**Timing analysis of pknots**. This figure builds on the previous figures and shows the amount of time the system spends in the various matrices. It can be seen that the system spends most of the time in the WHX matrix routine.

Amdahl's law states that the amount of speedup achievable is bounded by 1/((1-p)+p/s) where 'p' is the proportion of the original code where a speedup of 's' is obtained. Therefore, to obtain an upper bound for parallelism we set out to identify the section of the program where most of the time is spent. A timing analysis was performed on the scalar implementation, to clock each matrix filling function. The result of the timing analysis is shown in Figure 6.

From the Figure 6, it is clear that a bottleneck exists at the function that fills the WHX matrix. This may not necessarily be due to the function containing large amounts of work but simply the fact that it is located four levels deep into the nested configuration of the program. For that reason, the extremely low weights of the functions for VHX, ZHX, and YHX marks them out as unnecessary for attention. From this point onwards the parallelization efforts focus solely on this function which fills the WHX matrix, simply because of the ~90% contribution of this function towards the program's runtime.

**Figure 7:**
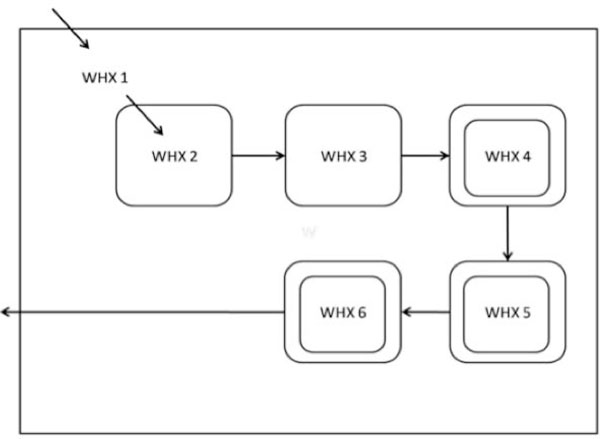
**WHX layout**. This figure describes the WHX matrix in more detail and shows the various sub-matrices. The number of borders indicate the level of iterations.

WHX consists of six major blocks of work. Each major block contains a large amount of comparisons between values from other matrices. A point to note is that there is no data dependency between the individual blocks. Figure 7 shows the WHX function with each block of work named WHXn where 'n' is in the range from 1-6. Each block is essentially a loop and the level of nesting is indicated by the number of borders. Note that maximum parallelization can be derived from WHX4 - WHX6 where as WHX1 does not contain loops and therefore in all subsequent parallelization efforts WHX1 is left untouched.

In addition to the individual loops (or work units) in the WHX matrix filling function being independent of each other, the loops themselves are each independent across iterations. We use this to our advantage in our implementations to improve the load balancing by performing SIMD-style loop parallelization.

### Code Parallelism (C-Par)

In the C-Par model, the PPE's primary task is managing synchronization between the SPEs. This model is known as function offloading and is the most popular way of parallelizing an existing application [11].

**Figure 8:**
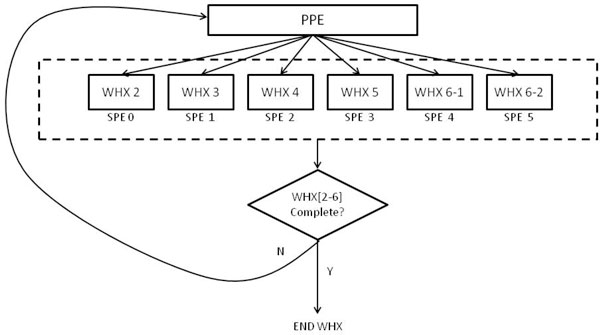
**C-Par implementation model**. This figure describes the work distribution under the C-Par implementation model. Since most of the computing time is spent in filling WHX matrix, the code is distributed to the various SPUs in the PS3.

In this implementation, data independence between the individual WHX blocks is exploited. Each block of work for filling WHX is now performed separately and the execution of each block is performed in parallel. The strategy is to run six SPEs, each handling one block of work, in essence running concurrently all the work blocks of WHX. Figure 8 illustrates this.

The memory requirement of the implementation is a major cause of concern. In particular, the thermodynamic information requires 70 MB of memory and since it has no predictable access pattern, neither the entire nor a portion of it can be copied to the SPEs.

**Figure 9:**
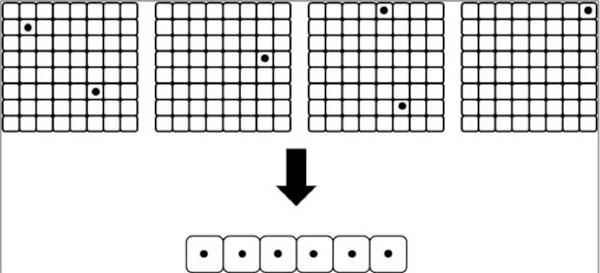
**Data packaging for transfer to SPEs**. This figure describes the optimization implemented in the data transfer from the PPU to the SPUs. It also highlights the intricacies of the IBM Cell platform.

Due to the memory constraints listed above, individual matrix elements are packaged together by the PPE into a contiguous block and fetched by the SPEs. In addition, all data transfers to the SPEs must be 16-bit aligned for transfer between 16 bytes to 16 kilobytes and also be perfectly aligned for transfers of 1, 2, 4, or 8 bytes. Figure 9 illustrates the data packaging technique to prepare the data for transfer from PPE to SPE. It involves assembling individual data values from different matrices into a single contiguous data package to satisfy processor-data alignment requirements.

On the negative side, the C-Par implementation didn't make effective use of the available computational power in the SPEs due to uneven number of loop iterations in matrix-filling functions and thus causing synchronization issues. In our next implementation, we aim to balance out the work load among the available SPEs.

### Data Parallelism (D-Par)

In this implementation, we switch to loop parallelization to improve load balancing among SPEs, exploiting the fact that there is no data dependency within each block of work (loop). This mitigates some effects of slowdown due to the non-constant number of iterations performed in each loop, which was the issue in C-Par implementation.

In this model, each SPU executes all the six functions sequentially for the same piece of input. CBE SDK was used to create entities known as SPE contexts [12] containing these functions.

**Figure 10:**
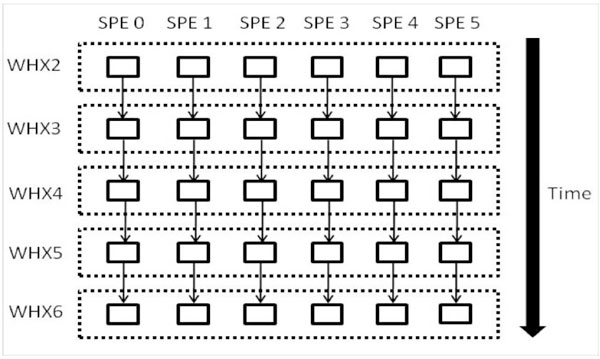
**D-Par parallelism implementation**. This figure describes the 2nd implementation method, D-Par. It also highlights the differences between earlier C-Par model especially with regards to the code and data swapping.

SPE contexts are an abstraction of SPE and contain the code and data to execute a work unit. During runtime, each SPE context is created by a dedicated PPE thread that stalls while the SPE executes. On completion, the SPE contexts is terminated by the corresponding PPE thread and the results are collected by the PPE. Figure 10 shows the D-Par implementation in a graphical way.

Through our experimental investigations, we found that SPE context creation and swapping is expensive on the IBM CBE. This is important for finer grain parallelization adopted in this implementation, as the functions are nested in loops and SPE contexts are swapped repeatedly. The overheads of setting up the parallel environment is amplified by the time complexity of the algorithm as well.

This created the issue of SPU initialization as different function codes needs to be swapped in and out of the SPU for each and every piece of data at various stages of the WHX{2-6} functions.

### Hybrid Parallelism (H-Par)

We created a 3rd implementation based on the experiences from the first two approaches. In particular, we wanted to avoid both SPE synchronization and the expensive SPE context switching.

The five SPE programs from the C-Par implementation was merged into a single SPE program to avoid performing repetitive SPE context creation and swapping and the SPEs are loaded & configured and run right at the start of the program. The SPE programs wait in busy loops until appropriate mailbox messages arrive from the PPE indicating the action is required. The WHX function has been rewritten to send these messages.

Synchronization is tweaked at the input parameter level to indicate to the SPE what set of computations it should perform for the current set of parameters. This approach has the advantage of loading a single function to all the SPEs while enabling to work on different functions based on the messages received.

We observed that the above H-Par implementation also showed signs of slowness for certain inputs. We further investigated and found that slow down happens in cases where the communication costs exceeds the actual computation to be performed by the SPEs.

Therefore, we introduced a tuning mechanism, during run-time, to decide on the locality of execution, i.e., whether to run on PPE or to distribute across SPEs. Thus whenever the input RNAs length is below a threshold value, the scheduler will run the code in the PPE and avoids invoking the SPEs, as the communication and synchronization costs are more than the computational gain achieved by executing the functions in the SPEs. For longer RNAs, SPEs are chosen for computation work loads.

**Figure 11:**
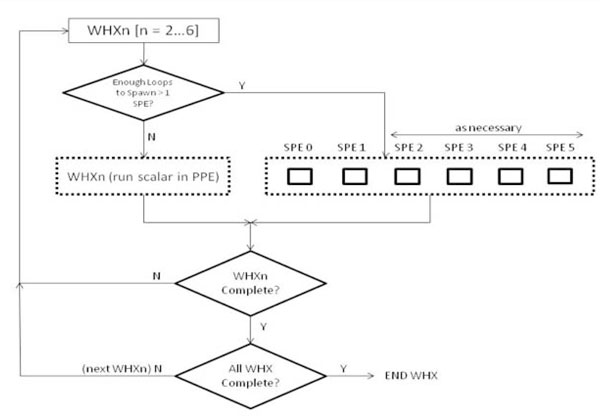
**H-Par parallelism implementation**. This figure describes the 3rd implementation method, H-Par. H-Par is the hybrid of the first two methods and adds run-time optimization decision primitives. This method takes into account the best execution location and also trades space with time.

In this H-Par implementation, the governing parameters are that each SPE should at least receive enough parameters to perform five iterations of the loop, and that the parallelization only happens when there is enough work to cater for at least two SPEs to run. Figure 11 shows the flow-chart of this implementation strategy.

### CBE scalar

For performance comparisons, we compiled a CBE scalar implementation not utilizing the SPEs. The scalar CBE implementation is obtained by compiling the pknots sources for the CBE PPE architecture, using the standard GCC tool-chain provided with the IBM Cell Software Development Kit v3.0.

### x86 scalar

For cross-platform performance comparisons, we compiled a x86-architecture based scalar implementation. The scalar implementation is obtained by compiling the pknots sources for the x86 architecture, using standard GCC tool-chain available in a Linux distribution.

## Results and discussions

In this section we elaborate the results obtained through our parallelization efforts and discuss the results based on two criteria - correctness verification and performance comparison. We used two hardware platforms for all our experiments. First, the Sony Play Station 3 was co-installed with a Linux Operating System with Linux kernel 2.6.x. Second, a x86 virtual machine using VMware Workstation 6.0 to run a virtual Linux Operating System with kernel 2.6.x. We used a virtual machine to intentionally limit the memory of the x86 platform to 256 MB (equivalent to the Sony Play Station 3) to make a fair comparison with the Cell processor.

Correctness in the context of this verification exercise is to ensure results are consistent across the parallel implementations with the original scalar version. In performance comparison, we compare the run-times of the parallel implementations with that of the scalar version running in a single-threaded mode on the CBE using only PPE and the x86 VM.

The results from our implementations were compared with the results from original pknots implementation using a set of real RNA sequences obtained from the Pseudo Base [13]. The lengths of the sequences obtained from this database are sufficiently diverse for correctness verification and have experimentally verified structures. The implementations recorded 100% correctness over the data set.

To begin with, we compare the results from the first two parallel implementations "C-Par" and "D-Par". The following Table 3 lists the run-times of the implementations for the set of RNA sequences.

**Table 3:** comparison of 'C-Par' and 'D-Par' implementations. This table details the run times for C-Par and D-Par implementations using RNA sequences of various lengths and also computes the speed ratio among them.

Sequence Length	Time (s)		
	**C-Par**	**D-Par**	**C:D**

21	10.52	8.72	1.206422018

22	13.82	11.32	1.220848057

23	17.97	14.88	1.20766129

25	29.65	23.52	1.260629252

30	88.54	71.33	1.241272957

34	188.65	147.32	1.280545751

38	362.93	289.69	1.252821982

40	493.86	399.51	1.236164301

40	493.9	397.71	1.241859646

41	573.51	467.41	1.226995571

42	661.65	541.78	1.221252169

46	1140.71	913.41	1.248847724

47	1297.22	1055.22	1.229336063

52	2373.26	1965.62	1.207384947

59	5055.58	4206.06	1.201975245

The effect of improving load balancing is seen in the superior performance of the D-Par implementation. However, the effect is not well pronounced as to suggest the first model was extremely unbalanced. An alternative interpretation is that the second model introduced balanced loads only when there was enough load to share between the SPEs, a tuning attribute of our implementation.

The importance of this result is the extent of slowdown due to improper parallelism that can occur on the CBE when the SPEs are underutilized. It implies the high overheads involved in getting an SPE to run, and hence significant loss of performance if they are run without actually offloading much work from the PPE. One source of such overheads in this dynamic programming implementation in particular is the DMA of small data fragments to operate on, as is the case before tuning when the program attempts to use SPEs for every minute amount of computation.

Secondly, we compare the D-par and H-par versions of the implementation. The following table shows the run-times of the 'D-par' and 'H-par' implementation for the same set of RNA sequences.

As can be observed from the above Table 4, 'H-par' out-performs 'D-par' significantly when the implementation is fused with intelligence to choose the location of execution for individual functions i.e., PPE for shorter RNA sequences and SPEs for higher work load.

**Table 4:** comparison of 'D-par' and 'H-par' implementations. This table details the run times for D-Par and H-Par implementations using RNA sequences of various lengths and also computes the speed ratio among them.

Sequence Length	Time (s)		
	**D-par**	**H-par**	**D:H**

21	8.72	1.66	5.253012048

22	11.32	2.13	5.314553991

23	14.88	2.74	5.430656934

25	23.52	4.39	5.357630979

30	71.33	12.89	5.533747091

34	147.32	27.61	5.335747917

38	289.69	54.76	5.29017531

40	399.51	75.20	5.312632979

40	397.71	75.43	5.272570595

41	467.41	87.63	5.333903914

42	541.78	101.65	5.329857354

46	913.41	177.10	5.157594579

47	1055.22	202.42	5.213022429

52	1965.62	377.72	5.203907656

59	4206.06	823.82	5.105557039

This result reinforces our earlier conclusion that for shorter RNA sequences it is counter-productive to introduce parallelism.

**Figure 12:**
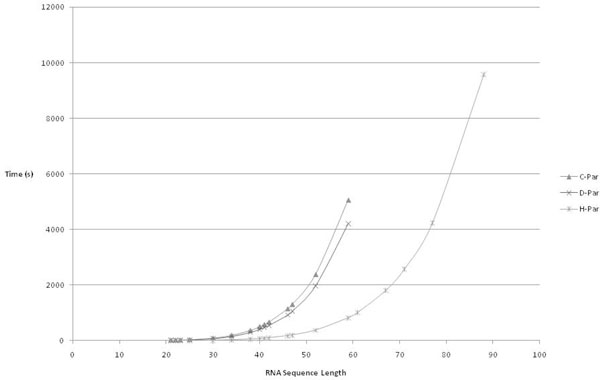
**Performances of 3 parallel implementations**. This figure plots the run-times of the 3 implementations against a standard set of input RNA sequences of various lengths. The X-axis plots the input data set and Y-axis plots the run-times. It can be seen that H-Par implementation has better performance when compared to the other two implementations.

Figure 12 plots the performance of all the above three parallel implementations. It can be observed the performance increases from the first to the third.

Thirdly, we compute two speedups to show the gain from parallelizing the DP algorithm the Cell architecture and also examine the scalar performance of Cell Vs and alternate architecture, i.e., x86.

The first speedup is obtained by comparing the performance of the H-par version of our implementation using both the PPE and SPEs against scalar version of the algorithm using only the PPE. This basically evaluates the contribution of parallelism against the scalar performance of the implementation on the CBE. From this point on we refer to "H-Par" implementation as "CBE parallel".

**Figure 13:**
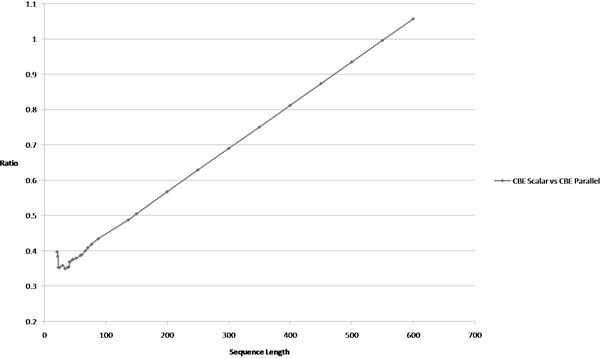
**CBE scalar vs CBE parallel speedup**. This figure shows the performance gain or speedup that is achieved by using parallelization in implementations. In this case, the speedup is on the same architecture and the scalar implementation uses only the scalar PPE processing cores of the IBM CBE whereas the parallel implementation uses all the available cores.

Figure 13 shows the speedup performance. We have analyzed the properties, trends and discuss them below.

For small RNAs, the nature of the dynamic programming algorithm will result in most of the computations involving shorter sub-sequences. These cases do not give rise to good opportunities to utilize the SPEs as there is too little work to be distributed. The CBE-parallel implementation is performing well to stay close to scalar performance for short strings, but ultimately there is additional overhead incurred in managing the SPEs. However, when RNA strings get longer, we see the parallel implementation start to close the gap as computation time starts to dominate the overall runtime by processing longer sub-sequences.

In Figure 13 the speedup advantage is an indication of the parallelism starting to take effect with longer RNA sequences. The data points are ratios and are obtained by dividing respective CBE-scalar runtimes over CBE-parallel runtime values.

The CBE-parallel implementation is configured to receive five iterations of a loop for computation and rejects utilizing the SPEs unless there is enough "rolls" of the loop to parallelize between at least two SPEs. For full utilization of six SPEs, each loop to be parallelized should have at least thirty iterations. For parallel performance to be substantial, the RNA sequence should be sufficiently long to be dominated by computations involving sub-sequences with lengths multiples of thirty. This shows that for RNA with lengths in the hundreds, the parallel performance is better than the scalar version.

Finally, in order to understand the suitability of the Cell architecture to a dynamic programming algorithm we compared the CBE scalar performance with an equivalent x86 performance of the algorithm on a virtual machine.

**Figure 14:**
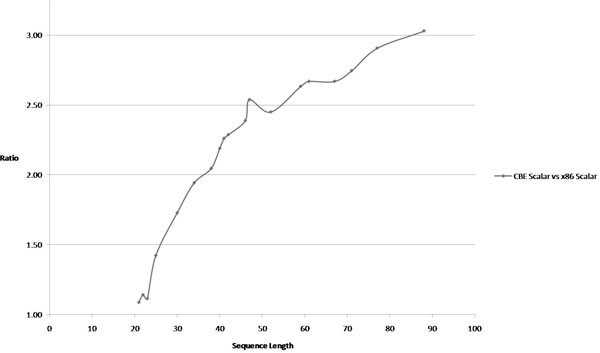
**scalar CBE performance vs x86 performance**. This figures shows the speedup performance between two scalar implementations that are implemented in two different CPU architectures.

The choice of x86 performance was mainly driven due to the fact that it is a commonly available machine, although one may expect that x86 with its sophisticated architecture will outperform CBE's PPE scalar performance. Figure 14 illustrates the speedup performance for this case.

We interpret the difference in performances among the two scalar implementations to be due to the difference of the hardware architectures - RISC Vs CISC. IBM Cell is a RISC based processor while the virtual machine is CISC based architecture.

Secondly, the limited/simple branch prediction hardware in the PPE/Cell, absence of Out-Of-Order execution unit, smaller cache sizes, less Instructions Per Cycle (IPC) could possibly slow down the CBE-Scalar implementation.

Thirdly, the dynamic programming algorithms for RNA secondary structure prediction uses deeply couple recursive data structures. This translates to high stack usage during run-time giving an advantage to CISC architectures when compared to RISC architectures. Also, Cell contains only hyper-threading while the PC had two independent processing cores.

Finally, as documented in [14] PPE is not suitable for performing computational work loads and more suited for control and command tasks. Therefore, running a full-fledged computational task like dynamic programming could have slowed down the computations as well.

## Conclusion

In this paper, we attempted to provide an high performance solution for handling RNA secondary structure prediction problem with pseudoknots. We selected IBM Cell broadband engine as the target platform and used Sony PS3 for implementation.

We considered dynamic programming approach proposed in [9] as a representative solution in all our implementations. We started by analyzing the existing scalar implementation to identify sections of code that can be safely parallelized. We designed 3 different implementations (C-Par, D-Par, H-Par) and analyzed the performances of them. In each of these strategies we exploited the embedded data and/or code parallelism in the DP. Our results conclusively show that a hybrid code & data parallelism approach as implemented in H-Par significantly outperforms the other two implementations that rely solely on code/data parallelism strategies.

Our results suggest a huge potential for parallelism in predicting secondary structures for long RNA sequences. The results also demonstrate that overheads due to parallelism may overshadow its effectiveness for shorter RNA sequences. One immediate and interesting extension is to use a high-end Cell-based processor such as Power × Cell 8i [10] in which addressable main memory is 16 GB in addition to other hardware enhancements. With such a high-end machine it is expected that the speedup will be more prominent even for shorter length sequences thus eliciting the unique advantage of using the CBE.

An alternate approach to address this RNA structure prediction problem is by designing customized algorithms that are suited solely for multi-core architectures as opposed to parallelizing the conventional DP solutions.

There are several conclusions which we draw from our current experience:

* Fine-grain parallelism, non-uniform data dependencies, locality in non-serial polyadic dynamic programming may not gain much in multi-core architectures. These concerns have been raised in [1] as well.

* The dynamic programming algorithm for RNA secondary structure [2] prediction contains nodes of comparison-intensive instructions (branching) as opposed to data compute and transformation. This puts the CBE in disadvantage.

* Dynamic Programming, due to its highly coupled recursive nature, is inherently suited for single-threaded execution and does not gain much from parallelization especially when sequence lengths are smaller.

* The approach of directly porting a scalar implementation to the CBE need not necessarily be the best way to introduce parallelism.

* Analysis of pknots may suggest the original scalar implementation was more favoring x86 architecture than using only the PPE in the IBM CBE architecture.

* Finally, we also believe that as more efficient compilers for CBE [15] are created, the true power of the CBE architecture will be evident.

## Competing interests

The authors declare that they have no competing interests.

## Authors' contributions

SPTK and BV were involved in the problem conception, algorithm design, result analysis and manuscript preparation. SPTK was involved in the technical design, software infrastructure setup and contributed to software development. BV was primarily involved in algorithm design. SSL was primarily involved in the software implementation and contributed to the manuscript preparations as well.
